# Functional dissociation in sweet taste receptor neurons between and within taste organs of *Drosophila*

**DOI:** 10.1038/ncomms10678

**Published:** 2016-02-19

**Authors:** Vladimiros Thoma, Stephan Knapek, Shogo Arai, Marion Hartl, Hiroshi Kohsaka, Pudith Sirigrivatanawong, Ayako Abe, Koichi Hashimoto, Hiromu Tanimoto

**Affiliations:** 1 Graduate School of Life Sciences, Tohoku University, Katahira 2-1-1, Miyagi, Sendai 980-8577, Japan; 2 Max-Planck Institut für Neurobiologie, D-82152 Martinsried, Germany; 3 Graduate School of Information Sciences, Tohoku University, Aramaki Aza Aoba 6-6-01, Aoba-Ku, Sendai 980-8579, Japan; 4 Department of Complexity Science and Engineering, Graduate School of Frontier Sciences, The University of Tokyo, 5-1-5 Kashiwanoha, Kashiwa-shi, Chiba-ken 277-8561, Japan

## Abstract

Finding food sources is essential for survival. Insects detect nutrients with external taste receptor neurons. *Drosophila* possesses multiple taste organs that are distributed throughout its body. However, the role of different taste organs in feeding remains poorly understood. By blocking subsets of sweet taste receptor neurons, we show that receptor neurons in the legs are required for immediate sugar choice. Furthermore, we identify two anatomically distinct classes of sweet taste receptor neurons in the leg. The axonal projections of one class terminate in the thoracic ganglia, whereas the other projects directly to the brain. These two classes are functionally distinct: the brain-projecting neurons are involved in feeding initiation, whereas the thoracic ganglia-projecting neurons play a role in sugar-dependent suppression of locomotion. Distinct receptor neurons for the same taste quality may coordinate early appetitive responses, taking advantage of the legs as the first appendages to contact food.

## Abstract (web summary)

Locating food sources is essential for the survival of animals. Here, the authors identify two functionally and anatomically distinct classes of sweet taste receptor neurons in Drosophila legs, involved in feeding initiation and sugar-dependent suppression of locomotion.

## Graphical abstract

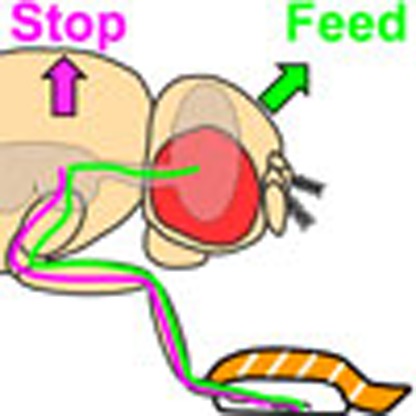

Animals rely on sensory systems for survival and reproduction. The gustatory system is important for finding and evaluating food sources, but can also have other roles. For *Drosophila* in particular, gustation is critical for mate and food selection. Mate selection requires specialized pheromone receptors in the legs of male flies^1^,^2^,^3^. Food selection is thought to involve the perception of multiple taste qualities, including bitter, sweet and salty tastants^4^,^5^,^6^,^7^,^8^,^9^, which promote or inhibit feeding.

In *Drosophila*, tastants are detected by taste hairs and taste pegs, most of which house multiple gustatory receptor neurons (GRNs)^10^, with each neuron tuned to a specific taste quality. Unlike mammals, where the tongue is the primary taste organ, insect taste hairs are distributed throughout the body. In particular, *Drosophila* GRNs can be found in the labellum, pharynx, tarsi, wing margins and female ovipositor^10^. So far, the functional significance of different taste organs is unclear. Notably, even though *Drosophila* GRNs are broadly distributed in the body, their central nervous system (CNS) projections are grouped according to taste organ. Projections from the pharyngeal, labellar and tarsal GRNs are located in the anterior, medial and posterior gnathal ganglia (GNG), respectively^7^,^11^,^12^,^13^. This implies that flies are able to differentially process gustatory information depending on stimulus location, and thus produce different behavioural outputs for the same stimulus^7^,^14^. In line with this, different sets of bitter taste receptor neurons in *Drosophila* are necessary for positional aversion and egg-laying preference^15^.

Feeding is a complex sequence of behaviours. In *Drosophila*, feeding is initiated by food detection and followed by locomotion arrest, extension of the proboscis and ingestion^16^. In addition, a recent study reported that sweet taste receptors in the pharynx promote ingestion prolongation^17^. It is therefore reasonable to assume that external taste organs are involved in earlier stages of appetitive behaviour. Once the decision to feed is made, the inhibition of other behaviours, such as locomotion, should occur. The recent identification of interneurons that influence the decision between feeding and moving in the *Drosophila* larva^18^ and adult^19^ helps to explain why these behaviours are mutually exclusive. However, the contribution of taste receptor neurons in deciding between these two behaviours remains unclear.

To better understand the role of different taste organs in appetitive behaviour, we targeted subsets of GRNs with the GAL4/upstream activating sequence (UAS) system^20^. We selected *Drosophila* lines^8^ expressing GAL4 under the control of various gustatory receptor promoters (*Gr-GAL4*) that have overlapping but distinct expression patterns in sweet taste receptor neurons. These lines differentially label the major *Drosophila* taste organs. To characterize the function of these different sets of GRNs, we developed an assay that allows the quantification of multiple feeding behaviours under conditions that resemble natural foraging. We then independently silenced subsets of GRNs and measured sugar preference in our assay. Our results show that sweet taste receptor neurons in the tarsi are essential for sugar choice. In the tarsi, we identified two anatomically distinct populations of sweet taste receptor neurons that are involved in different appetitive behaviours. Taken together, our results highlight a functional dissociation between and within taste organs of *Drosophila*.

## Results

### Hungry flies show rapid and robust sugar preference

**Figure 1:**
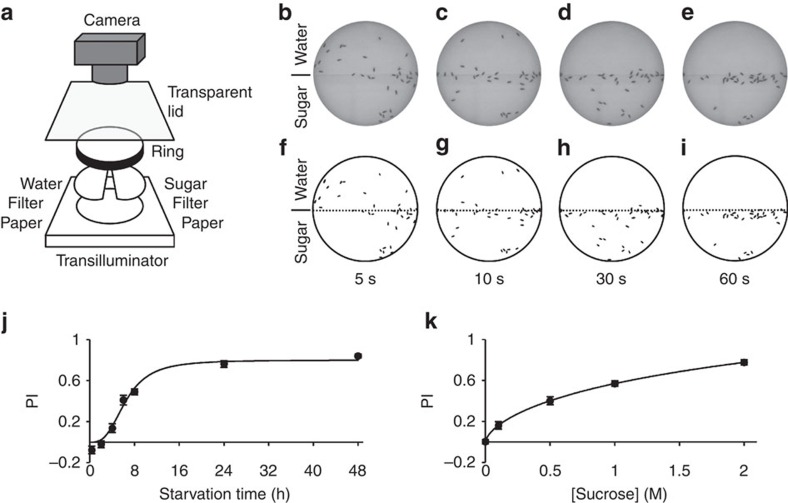
The sugar preference assay. (**a**) Schematic of the experimental set-up used to measure sucrose preference. (**b**–**e**) Examples of the distribution of starved (36 h) Canton S flies in the circular arena at various time points (5–60 s) after introduction. A 2-M sucrose solution was used for the sucrose side (lower semicircle). (**f**–**i**) Same images as in **b**–**e** after applying a suitable threshold with the Fiji software. Identified particles are in black. (**j**) Starvation dependency of sucrose PI of Canton S flies. A 2-M sucrose solution was used. *n*=8–12 per starvation interval. (**k**) Concentration dependency of sucrose PI of Canton S flies. Flies were starved to 20% mortality (average starvation time 40 h). *n*=11–15 per concentration. Results are means±s.e.m.

We developed a simple behavioural paradigm, the sugar preference assay, to quantify early appetitive responses of fruit flies (Fig. 1). In the sugar preference assay, we introduced freely walking flies into a circular arena, where they were allowed to choose between two sides: one with sugar and one without (Fig. 1a). Fly behaviour was video-recorded from above, allowing subsequent quantification of position and locomotion. We used the wild-type Canton S strain to characterize the assay. Starved flies showed quick and robust responses to sucrose (Fig. 1b–e). We calculated preference indices (PIs) by automatically counting and subtracting the fly numbers on the sugar and non-sugar sides in a given video frame (Fig. 1f–i). Plotting PI over time revealed that wild-type flies chose the sugar side within the first 20–30 s, with PIs reaching a plateau thereafter (Supplementary Fig. 1). We therefore pooled the data between 30 and 60 s for each experiment, and simply refer to them as preference or PIs. PIs were strongly dependent on the degree of starvation and sugar concentration. In particular, the average PI increased steadily between 2 and 8 h of starvation, and reached a plateau between 24 and 48 h (Fig. 1j and Supplementary Fig. 1a). Similarly, the PI rose with increasing sucrose concentration for long-starved flies (∼20% mortality; Fig. 1k and Supplementary Fig. 1b). Given the high signal-to-noise ratio, we chose the long starvation/high sugar concentration conditions for the following experiments.

### Sweet GRNs in the legs are required for sugar preference

**Figure 2:**
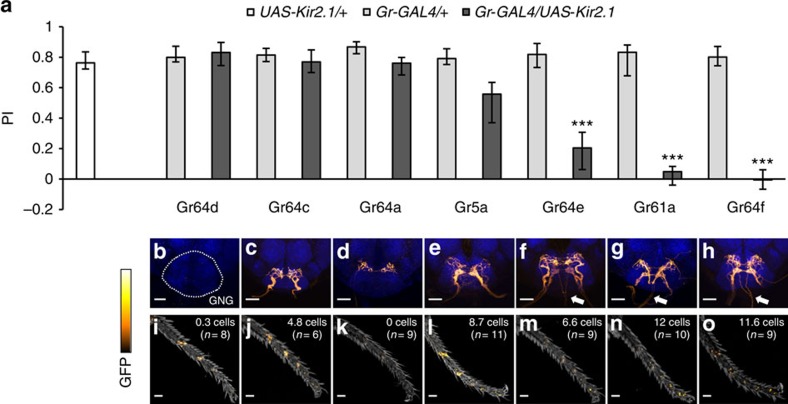
Blocking specific subsets of sweet taste receptor neurons differentially affects sucrose preference. (**a**) Requirement of different sweet taste receptor neurons for sucrose preference. Electrically silencing sweet taste receptor neurons in *Gr61a-GAL4*, *Gr64e-GAL4* and *Gr64f-GAL4* with constitutively active *UAS-Kir2.1* impaired 2-M sucrose PI compared with genetic controls (Kruskal–Wallis test; Dunn's post test; ***P<0.001). Driving *UAS-Kir2.1* by all other tested *Gr-GAL4* lines did not significantly impair sucrose preference (*P*>0.05). Sucrose preference of flies with silenced sweet taste receptor neurons using *Gr61a-GAL4* and *Gr64f-GAL4* was indistinguishable from zero (Wilcoxon signed-rank test, *P*>0.05). *n*=12–23 per group. Results are medians, error bars indicate the first/third quartile. (**b**–**h**) Expression patterns of *Gr-GAL4* lines in the GNG (*UAS-mCD8::GFP*, orange; Synapsin (ubiquitous synaptic marker), blue). Partial projections, scale bars, 40 μm. Note inputs from the VNC via the cervical connective (arrows) only in the three *Gr-GAL4* lines with impaired sucrose preference. (**i**–**o**) Expression patterns of *Gr-GAL4* lines in foreleg tarsi (*UAS-mCD8::GFP*, orange). Total cell numbers (mean) of all tarsal segments are reported. Strong signals in the joints are autofluorescence. Scale bars, 40 μm, *n*=6–11.

To examine the role of different taste organs in appetitive behaviour, we next chose to silence subsets of sweet taste receptor neurons. We selected seven *Gr-GAL4* lines labelling GRNs that express sets of homologous sweet taste receptors (Fig. 2a)^21^. We drove expression of Kir2.1, an inward-rectifying potassium channel^22^, to electrically silence sweet taste receptor neurons by the specific Gr-driven GAL4. We then tested flies for sugar preference (Fig. 2a). We found that sugar preference was highly variable among the drivers, ranging from normal preference to complete impairment of sugar preference. The blockades with *Gr61a-GAL4* and *Gr64f-GAL4* abolished sucrose preference, and blocking with *Gr64e-GAL4* caused a statistically significant decrease. To better understand the observed differences, we examined the expression patterns of all GAL4 lines in the GNG and forelegs (Fig. 2b–o). Our detailed anatomical analyses are consistent with a recent study^23^ and highlight three key findings. First, expression in the labellar nerve did not always yield strong sugar preference impairment (*Gr64c-GAL4* and *Gr5a-GAL4* in Fig. 2). Second, *Gr64a-GAL4* specifically labelled pharyngeal GRN terminals (Fig. 2d,k) but showed normal preference. Detailed anatomical analysis revealed that *Gr64a-GAL4* and *Gr64f-LexA* co-labelled four cells in the labral sense organ (LSO), but did not label the ventral or dorsal cibarial sense organs (Supplementary Fig. 2), in line with a recent study^17^. Therefore, these LSO GRNs are not critical for sugar choice. Third, only *Gr61a-GAL4* and *Gr64f-GAL4*, which labelled the maximum number of GRNs in the legs (12 cells, Fig. 2n,o) showed abolished sugar preference on blockade. Taken together, our results suggest that sweet taste receptor neurons in the legs, but not the labellum or LSO, are critical for sugar preference in *Drosophila*.

### Two anatomically distinct classes of tarsal sweet GRNs

**Figure 3:**
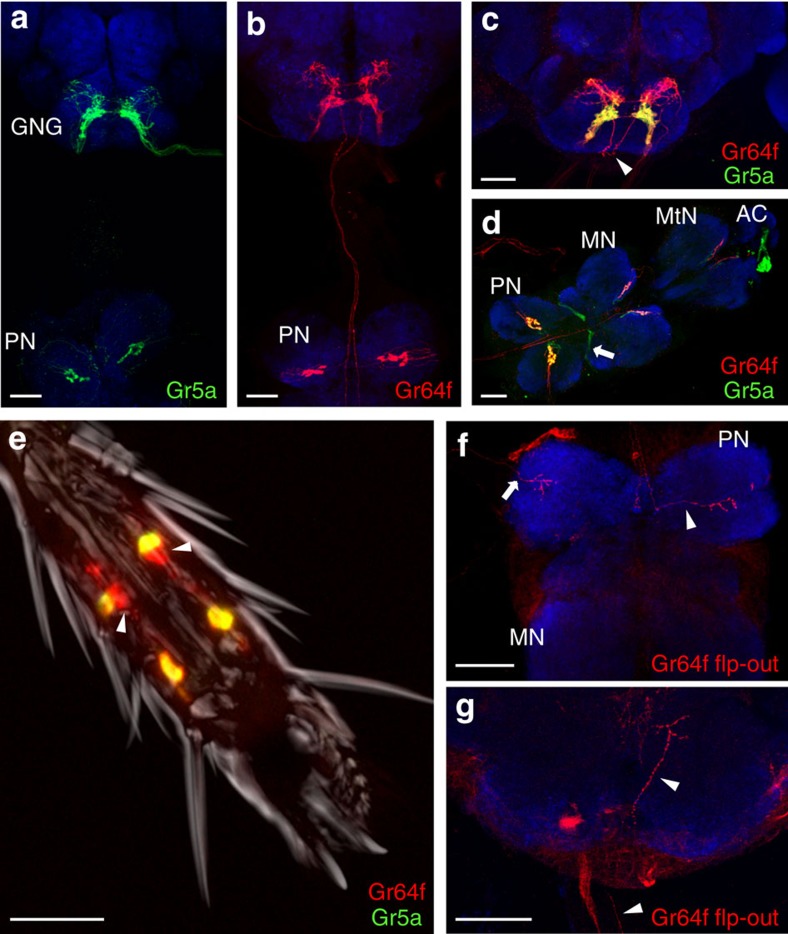
Two classes of anatomically distinct sweet taste receptor neurons in the legs of *Drosophila*. (**a**,**b**) Expression patterns of *Gr5a-GAL4* (**a**) and *Gr64f-GAL4* (**b**). (**c**–**e**) Double labelling of *Gr64f-GAL4* with *UAS-mCD8::RFP* (red) and *Gr5a-LexA* with *LexAop-mCD8::GFP* (green) in the GNG (**c**), pro-, meso-, metathoracic ganglia (PN, MN and MtN) and abdominal centre (AC) of the VNC (**d**) and fifth tarsal segment of the foreleg (**e**). Expression in the GNG and VNC is widely overlapping (yellow). Note non-overlapping expression in atGRNs (arrowheads in **c** and **e**; red) and projections from the wings (arrow in **d**; green). (**f**,**g**) Single GRN flp-outs in the VNC (**f**) and GNG (**g**) (red). Note the distinct projections from a stGRN (**f**, arrow) and an atGRN (**f**,**g** arrowheads). Synapsin (ubiquitous synaptic marker) staining shown in blue. Partial projections, scale bars, 20 μm (**e**) or 40 μm (**a**–**d**,**f**,**g**).

Strikingly, blocking with *Gr5a-GAL4* resulted in a modest PI decrease, despite its broad expression pattern (Fig. 2). *Gr5a-GAL4* labels all sweet taste receptor neurons in labellar taste hairs^8^ and most sweet taste receptor neurons in the legs. In contrast, *Gr64f-GAL4*, which labels only a few additional cells in the legs, showed no sugar preference (Fig. 2a) on blockade. To understand this difference, we examined the differences in *Gr5a-GAL4* and *Gr64f-GAL4* expression in greater detail (Fig. 3). Both lines labelled inputs from the labellum and legs (Fig. 3a,b). However, only *Gr64f-GAL4* marked ascending fibres from the ventral nerve cord (VNC) to the cervical connective and the posterior GNG (Fig. 3a,b).

To better contrast differentially labelled cells, we used *Gr5a-LexA* and *Gr64f-GAL4* to drive different reporters in the same fly. Overall, both drivers showed highly overlapping expression (Fig. 3c–e; yellow). The GRNs labelled in both *Gr5a-GAL4* and *Gr64f-GAL4* terminate in a given thoracic neuromere, while a few additional GRNs in the fifth tarsal segment of *Gr64f-GAL4* project directly to the GNG through the cervical connective (Fig. 3c–e). Despite the lesser requirement of *Gr5a-GAL4*-labelled cells for sugar preference (Fig. 2), the number of common tarsal GRNs is more than twice as many as the ascending cells (9–10 versus 2–4 cells). Single-cell analysis^24^ with *Gr64f-GAL4* revealed both cell populations in the CNS (Fig. 3f,g). Interestingly, the ascending cells displayed axon collaterals in the VNC (Fig. 3f), which intermingled with the VNC terminals of the other tarsal GRNs. These results show that sweet taste receptor neurons in the legs are classified into two anatomically distinct groups: the ascending tarsal GRNs (atGRNs) and the segmental tarsal GRNs (stGRNs).

### atGRNs are required for feeding initiation

**Figure 4:**
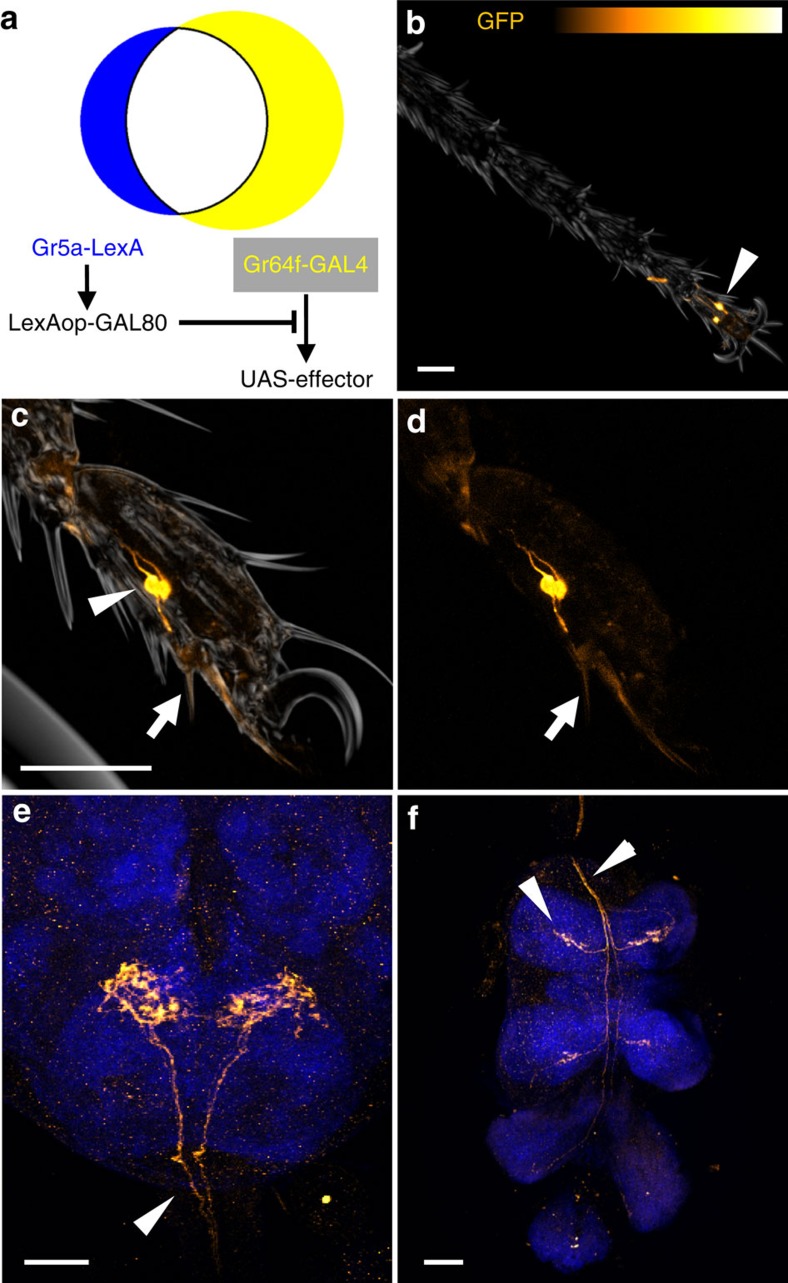
Anatomy of atGRNs in the periphery and CNS. (**a**) Subtraction of *Gr5a-LexA* expression from *Gr64f-GAL4* with *LexAop-GAL80* restricts UAS-effector expression to non-overlapping GRNs. (**b**–**f**) Expression pattern of Gr(64f–5a) in the foreleg (**b**), the fifth tarsal segment (**c**,**d**), the GNG (**e**) and the VNC (**f**). *UAS-mCD8::GFP*, orange; Synapsin, blue; scale bars, 40 μm. (**b**–**d**) Only 1–2 pairs of atGRNs were labelled in the forelegs. atGRNs have cell bodies in the fifth tarsal segment (arrowheads) and innervate a distinct pair of ventral sensilla in the distal part of the foreleg (arrows). (**e**) Input to the GNG in Gr(64f–5a) flies derives from the VNC (arrowhead). GFP was not detected in fibres projecting from the labellum. (**f**) Tarsal fibres project to the VNC and ascend via the cervical connective to the GNG (atGRNs, arrowheads).

Because atGRNs were labelled in all *Gr-GAL4*s that caused significant sugar preference impairments (Fig. 2), we hypothesized that they are crucial for sugar detection, and sought to manipulate them more specifically. We used *Gr5a-LexA* to express GAL80, which binds to GAL4 and suppresses its ability to activate transcription, to silence *Gr64f-GAL4* in cells that co-express *Gr5a-LexA*. This approach genetically ‘subtracted' labellar and stGRN expression in *Gr64f-GAL4* (Fig. 4a), allowing visualization of cells that express *Gr64f-GAL4* alone. For brevity, we refer to this genetic subtraction approach as Gr(64f–5a) hereafter. Gr(64f–5a) specifically marked the atGRNs (Fig. 4b–f) and four cells in the LSO (Supplementary Fig. 3), and revealed that the atGRNs innervate a pair of short, distal-most ventral taste hairs beneath the claws (Fig. 4c,d). These are likely the taste hairs recently termed 5V1 (ref. 25) or f5v (ref. 4).

**Figure 5:**
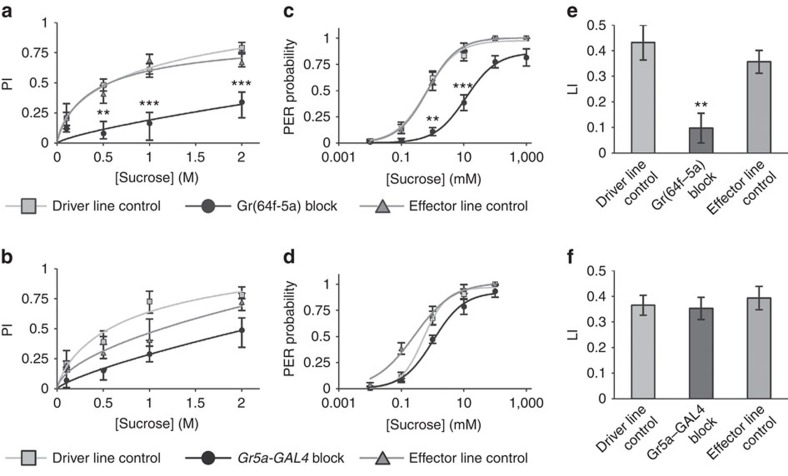
atGRNs are involved in multiple appetitive behaviours. (**a**) Sucrose PI was significantly impaired at 0.5, 1 and 2 M on blocking Gr(64f–5a) cells (Kruskal–Wallis test; Dunn's post test; **P<0.01; ***P<0.001). *n*=9–21 per group. Driver line control *Gr64f-GAL4/+*, Gr(64f–5a) block *Gr5a-LexA/+; Gr64f-GAL4/LexAop-GAL80; UAS-Kir2.1/+*, effector line control *Gr5a-LexA/+; LexAop-GAL80/+; UAS-Kir2.1/+*. (**b**) Sucrose preference was not significantly impaired on blocking *Gr5a-GAL4* cells (Kruskal–Wallis test; Dunn's post test; *P*>0.05). *n*=11–21 per group. Driver line control *Gr5a-GAL4/+*, *Gr5a-GAL4* block *Gr5a-GAL4/UAS-Kir2.1*, effector line control *UAS-Kir2.1/+*. (**c**) Tarsal PER was significantly impaired at 1 and 10 mM on blocking Gr(64f–5a) cells (Kruskal–Wallis test; Dunn's post test; **P<0.01; ***P<0.001). *n*=20–50 flies per group. (**d**) PER after tarsal stimulation was not significantly impaired on blocking *Gr5a-GAL4* cells (Kruskal–Wallis test; Dunn's post test; *P*>0.05). *n*=35–58 flies per group. (**e**) Short-term appetitive olfactory memory was abolished on blocking subtraction cells (LI, learning index; one-way analysis of variance (ANOVA); Bonferroni's multiple comparison test; **P<0.01). *n*=11–12 per group. (**f**) Short-term appetitive olfactory memory was unaffected on blocking *Gr5a-GAL4* cells (one-way ANOVA; Bonferroni's multiple comparison test; *P*>0.05). *n*=9–10 per group. Results are medians, with the error bars indicating the first/third quartile (**a**,**b**) or means±s.e.m. (**c**–**f**).

We next used Gr(64f–5a) to drive Kir2.1 expression, and addressed the requirement for the atGRNs in sugar preference. We found a strong, albeit incomplete, reduction in sucrose preference across the concentration range (Fig. 5a), suggesting that atGRNs are not tuned to specific sugar concentrations. In contrast, blocking the labellar and stGRNs with *Gr5a-GAL4* yielded a more modest effect (Fig. 5b). The requirement for Gr(64f–5a) cells is consistent with the strong preference impairments with *Gr61a-GAL4*, *Gr64e-GAL4* and *Gr64f-GAL4*, which all label the atGRNs (Fig. 2). Furthermore, subtracting *Gr5a-LexA* from *Gr61a-GAL4* and blocking the Gr(61a–5a) cells impaired preference to the same extent as the Gr(64f–5a) blockade (Supplementary Fig. 4). *Gr43a-GAL4* also labels atGRNs, but blocking with this line did not alter sucrose preference (Supplementary Fig. 5). However, *Gr43a-GAL4* also labels multiple cells outside the three main taste organs, which are not labelled by *Gr64f-LexA* (Supplementary Fig. 5), and these off-target cells can improve appetitive performance^26^.

To segregate the contribution of tarsal GRNs from that of the other organs, we used the proboscis extension reflex (PER) assay with tarsal stimulation^7^,^27^. In accordance with the sugar preference results, PER in response to sucrose solutions of varying concentrations was significantly impaired when atGRNs were blocked with Gr(64f–5a) (Fig. 5c). In contrast, blocking stGRNs with *Gr5a-GAL4* caused a smaller but statistically not significant decrease of PER (Fig. 5d). Interestingly, PER was abolished when the labellum of the *Gr5a-GAL4/UAS-Kir2.1* fly was stimulated with a 200-mM sucrose solution (Supplementary Fig. 6a), whereas the blockade with Gr(64f–5a) did not significantly alter PER on stimulation of the labellum with sucrose (Supplementary Fig. 6b). Taken together, these results support the idea that atGRNs are important for initiating feeding after encountering food.

Apart from driving early appetitive responses, sugar ingestion acts as a reward and induces appetitive memory^28^. Given the important role of atGRNs in feeding initiation, we next considered the significance of different subsets of sweet taste receptor neurons for sugar reward. We used sucrose as a reward, and blocked distinct sweet taste GRNs with Gr(64f–5a) and *Gr5a-GAL4*. Blocking the atGRNs greatly reduced short-term olfactory memory (Fig. 5e), while blocking the labellar GRNs and the stGRNs had no significant effect (Fig. 5f). We excluded defects in olfactory perception and/or choice by testing the same flies in aversive memory with the same odours (Supplementary Fig. 7). These results suggest that the early appetitive responses controlled by atGRNs are important for sugar reward. The stGRNs should contribute to other aspects of the sugar response.

### stGRNs are required for locomotion suppression

**Figure 6:**
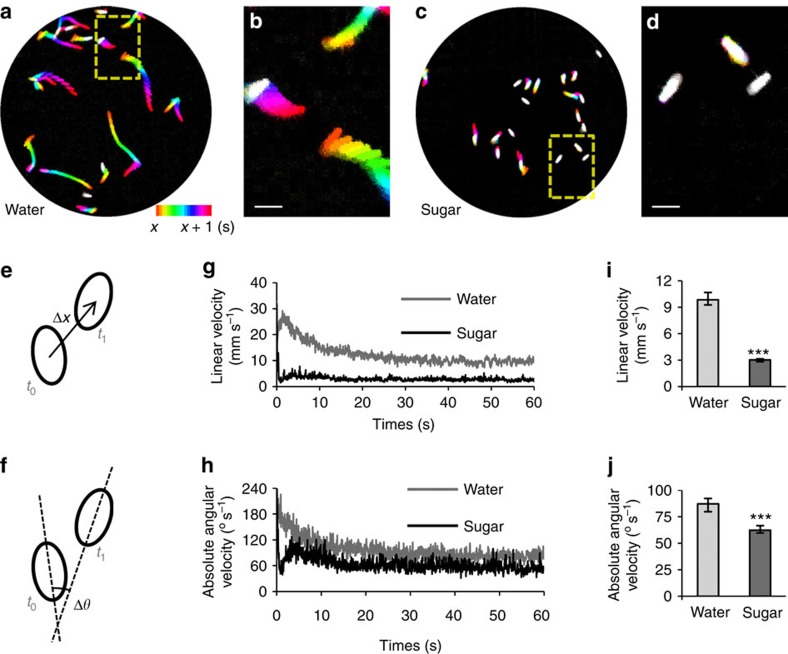
Sugar suppresses the locomotor activity of hungry flies. (**a**–**d**) Locomotion behaviour of starved wild-type flies in the absence (**a**,**b**) or presence (**c**,**d**) of 2 M sucrose during a 1-s interval (*x*=55 s in **a** and 57 s in **b**). Flies are colour-coded according to time. Blow-up panels show locomotion of selected flies in the absence (**b**) and presence (**d**) of sugar. Scale bars, 3 mm. (**e**,**f**) The linear (**e**) and angular (**f**) velocity of flies were calculated by fitting ellipses to flies and computing the changes in the centroid position (Δ*x*) and the major axis' angle (Δ*θ*), respectively. (**g**,**h**) Time series of the average linear velocity (**g**) and the average absolute angular velocity (**h**) of all flies in the absence (grey; *n*=12) or presence (black; *n*=11) of 2 M sucrose. (**i**,**j**) Average linear velocity (**i**) and average absolute angular velocity (**j**) of all flies between 30 and 60 s of the experiment in the absence (light grey; *n*=12) or presence (dark grey; *n*=11) of 2 M sucrose. Sucrose significantly reduced both linear and angular velocity (Mann–Whitney *U*-tests; ***P<0.001). Results are medians, with the error bars indicating the first/third quartile.

When we blocked stGRNs and tested for sugar preference (Figs 2 and 5), we noted that the flies tended to be restless even on sugar in contrast to control flies that exhibited very little movement. We therefore decided to quantify fly locomotion in response to sugar. To avoid the complication of a binary choice, the entire arena was covered with either water or sugar for locomotion measurements (Fig. 6a–d).

To quantify locomotor activity, we developed software to detect flies in each video frame and to calculate the linear and absolute angular velocity of each fly between consecutive frame pairs (Fig. 6e,f; Methods). These two behavioural variables represent sugar-induced arrest of walking and turning, respectively. We evaluated the accuracy in assigning fly identity in two frames by examining more than 1,000 fly pairs from random frame pairs and videos. Identity was correctly assigned in the majority of the cases (error rate 0.7%). The orientation and position of the flies were also accurately estimated, with the average errors in body axis and centroid position being 2.2±0.2° and 0.108±0.006 mm (∼5% of body length).

Locomotion of wild-type (Canton S) flies was greatly reduced in the presence of 2 M sucrose (Fig. 6a–d). On water, the average walking and turning velocity were initially high, dropped gradually and stabilized after 20–30 s (Fig. 6g,h). The high locomotor activity was presumably because of a startle response caused by fly introduction into the arena. In the presence of sugar, flies showed significantly lower activity throughout the experiment (Fig. 6g,h). To quantify the sugar-induced suppression in locomotion at the steady state, we pooled the average linear velocity and the average absolute angular velocity from 30 to 60 s (Fig. 6i,j). Both of these behavioural variables were significantly reduced in the presence of sugar.

**Figure 7:**
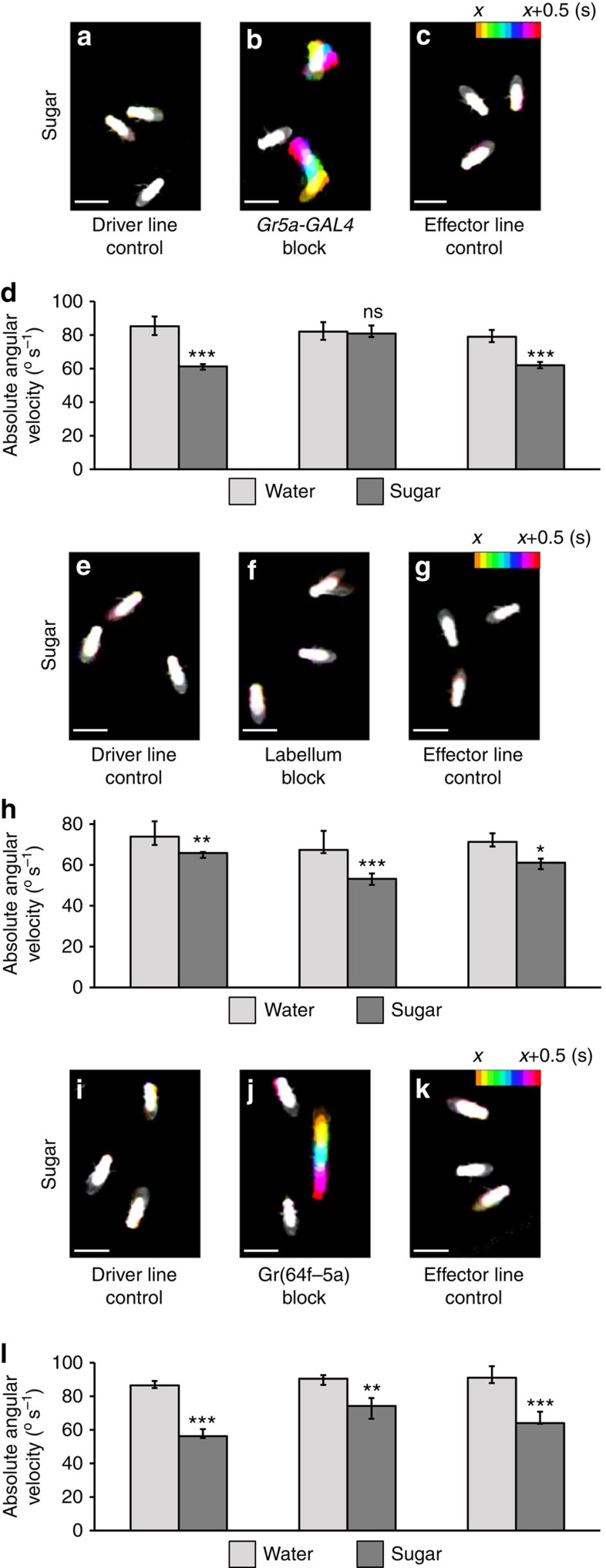
stGRNs are required for sugar-dependent turning suppression. (**a**–**c**) Examples of locomotion of (**a**) *Gr5a-GAL4/+*, (**b**) *Gr5a-GAL4/UAS-Kir2.1* and (**c**) *UAS-Kir2.1/+* flies during half-second intervals (*x*=46.4 s in **a**, 55.5 s in **b** and 50 s in **c**). Flies are colour-coded according to time. Scale bars, 3 mm. (**d**) Average absolute angular velocity of all flies between 30 and 60 s of the experiment in the absence (light grey, *n*=15–16) or presence (dark grey; *n*=16–17) of 2 M sucrose. Sucrose significantly reduced angular velocity for genetic controls, but not for the experimental group (Mann–Whitney *U*-tests; ***P<0.001; not significant *P*>0.05). (**e**–**g**) Examples of locomotion of (**e**) *Gr5a-GAL4/+; tub>GAL80>/+*, (**f**) *Gr5a-GAL4/otd-nls-FLPo; tub>GAL80>/UAS-Kir2.1* and (**g**) *otd-nls-FLPo/+; UAS-Kir2.1/+* flies during half-second intervals (*x*=56 s in **e**, 51.8 s in **f** and 31.6 s in **g**). Flies are colour-coded according to time. Scale bars, 3 mm. (**h**) Average absolute angular velocity of all flies between 30 and 60 s of the experiment in the absence (light grey, *n*=13–14) or presence (dark grey; *n*=13–14) of 2 M sucrose. Sucrose significantly reduced angular velocity for all groups (Mann–Whitney *U*-tests; ***P<0.001; **P<0.01; *P<0.05). (**i**–**k**) Examples of locomotion of (**e**) *Gr64f-GAL4/+*, (**f**) Gr(64f–5a) block and (**g**) the associated effector line control flies during half-second intervals (*x*=49.5 s in **i**, 47.6 s in **j** and 51.7 s in **k**). Flies are colour-coded according to time. Scale bars, 3 mm. (**l**) Average absolute angular velocity of all flies between 30 and 60 s of the experiment in the absence (light grey, *n*=8–11) or presence (dark grey; *n*=9–11) of 2 M sucrose. Sucrose significantly reduced angular velocity for all groups (Mann–Whitney *U*-tests; ***P<0.001; **P<0.01). Results are medians, with the error bars indicating the first/third quartile.

Strikingly, blocking the cells in *Gr5a-GAL4* abolished the sugar-induced suppression of turning (Fig. 7d). The same cells were also required for sugar-induced walking suppression (Supplementary Fig. 8a). As *Gr5a-GAL4* labels both the labellum and stGRNs, we introduced *otd-nls-FLPo* (ref. 29), which is expressed only in the head^30^, and *tub>GAL80>* to limit transgene expression to the labellum. In contrast to the *Gr5a-GAL4/UAS-Kir2.1* flies, flies with silenced labellar neurons reduced their locomotion on sugar (Fig. 7h and Supplementary Fig. 8b), suggesting that stGRNs are required for locomotion inhibition upon sugar detection. In contrast, flies with blocked atGRNs showed sugar-induced suppression of walking and turning (Fig. 7l and Supplementary Fig. 8c), despite their critical role in feeding initiation (Fig. 5). Taken together, our results highlight a functional dissociation of sweet taste receptor neurons in the tarsus. Both atGRNs and stGRNs are required for early appetitive responses, but the atGRNs are critical for feeding initiation, whereas the stGRNs are involved in the locomotion suppression upon food encounter.

### Sugar preference integrates both early appetitive responses

**Figure 8:**
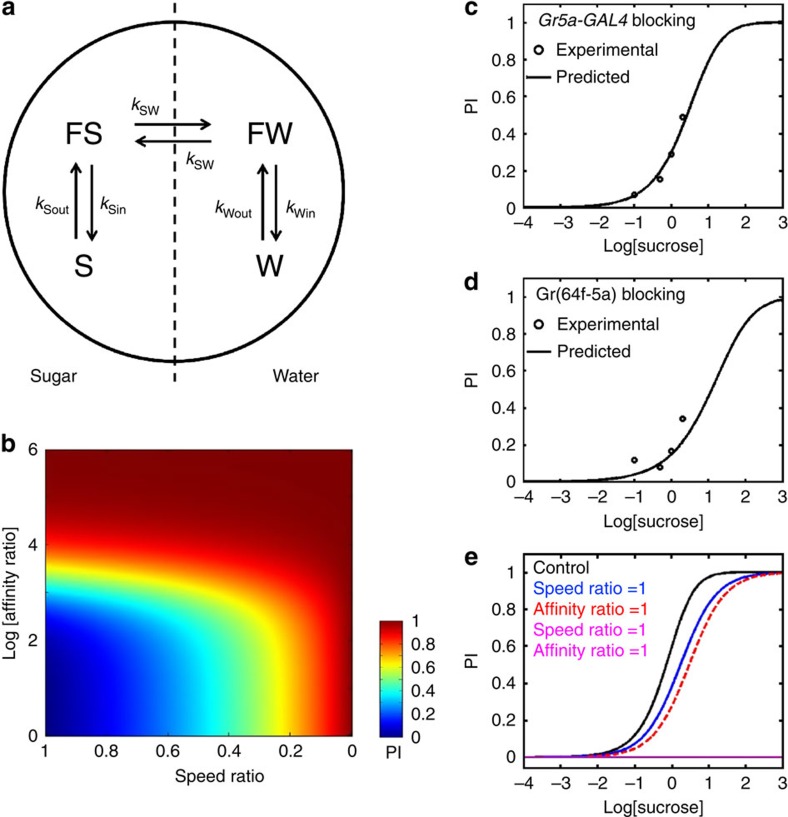
Mathematical model of sugar preference. (**a**) Schematic of the model. Flies can transition between ‘free to walk' states (FS and FW) and ‘feeding' states (S and W) on the sugar and water sides of the sugar preference assay. All transitions are reversible (bidirectional arrows) and controlled by constant transition rates *k*. (**b**) Effect of the sugar/water speed ratio and sugar/water affinity ratio on sugar PI. (**c**,**d**) Quantitative predictions of the model for the *Gr5a-GAL4* (**c**) and Gr(64f–5a) (**d**) blockades and comparison with the corresponding experimental data (open circles). (**e**) Effect of loss of sugar-induced locomotion suppression (blue), loss of sugar affinity (red) and both (magenta) on the PIs of control flies (black).

To mechanistically understand how different sensory inputs are integrated to drive choice in the sugar preference assay (Fig. 1), we devised a dynamic-state transition model to mathematically predict PIs. We assigned flies to the mutually exclusive ‘free to walk' and ‘feeding' states on the sugar and water sides, for a total of four states (Fig. 8a). By definition, only ‘free to walk' flies can cross the border between sugar and water (FS and FW in Fig. 8a). ‘Free to walk' flies can also transition to a ‘feeding' state on the same side (S and W in Fig. 8a). Transitions between states are controlled by certain rates (constants *k* in Fig. 8a).We equated the transition rates between the two ‘free to walk' states to the linear velocities on sugar and water (Supplementary Fig. 8) and refer to their sugar/water ratio as ‘speed ratio'. We also reasoned that the ratio of the two transition rates for the ‘feeding' and the corresponding ‘free to walk' state (*k*_in_/*k*_out_ in Fig. 8a) depends on stimulus affinity. Therefore, we derived the transition ratios from the PER data (Fig. 5c,d). For brevity, we will refer to the ratio of sugar/water affinities as ‘affinity ratio'.

We first examined how the affinity and speed ratios influence PI in the sugar preference assay by simulating it for different values of the two parameters (Fig. 8b). In line with our neuronal silencing results (Fig. 5a,b), sugar preference depended on both parameters, but the dependence on affinity was greater. For quantitative predictions, we first determined the free parameters of the model with data from genetic controls. Consequently, we inputted experimental data of PER and sugar-dependent locomotion suppression (from Fig. 5c,d and Supplementary Fig. 8) into our model to predict the dose–response curves of preference for the *Gr5a-GAL4* and Gr(64f–5a) blockades (Fig. 8c,d). The outputs of the model showed good agreement with the experimental data, demonstrating that the model can make good quantitative predictions. Finally, we investigated how wild-type sugar preference is affected when sugar affinity and/or locomotion suppression are lost (affinity and/or speed rations of one, Fig. 8e). Again, loss of affinity had a greater effect than lack of locomotion suppression. Intriguingly, sugar preference was abolished only if both responses were lost. This correctly predicts the lack of sugar preference observed with *Gr61a-GAL4* and *Gr64f-GAL4* silencing (Fig. 2) and highlights the nonlinear interaction between atGRNs and stGRNs. In conclusion, our model makes accurate predictions based on few assumptions and therefore captures key aspects of choice behaviour.

## Discussion

Insects have multiple taste organs distributed throughout the body, but the functional significance of this organization has been unclear. We therefore utilized sweet taste receptor neurons in *Drosophila melanogaster* as a model to study this question and showed that GRNs in different taste organs, or even within the same organ, have functional specialization. Sweet taste receptor neurons in the legs, but not the labellum or LSO, are necessary for the early appetitive response to sugar (Fig. 2). Using a genetic subtraction approach (Fig. 4) and three different paradigms of appetitive behaviour (Fig. 5), we showed that the atGRNs, a small subset of tarsal GRNs, are critical for feeding initiation. The other subset of tarsal sweet receptors that terminate in the VNC, stGRNs, control locomotion arrest on encountering sugar (Figs 6 and 7). We devised a model of sugar preference, which predicts that both appetitive responses influence sugar preference and that loss of both is required to generate ‘sugar-blind' flies (Fig. 8). Taken together, our data demonstrate that the atGRNs and stGRNs are preferentially tuned to distinct facets of the early appetitive response. However, more detailed characterization of the receptor–ligand relationships in the legs with regards to sugar choice should await further experimentation.

The roles of atGRNs and stGRNs are unlikely to be mutually exclusive. For example, the atGRNs may affect locomotion directly with collaterals in the VNC (for example, Figs 3f and 4f) and/or indirectly through PER, as proboscis extension was recently shown to negatively regulate walking^19^. On the other hand, blocking stGRNs with *Gr5a-GAL4* gave trends of impairment in sugar preference and PER (Figs 2 and 5). The involvement of stGRNs in these behaviours is consistent with previous studies using activation and inhibition with *Gr5a-GAL4* (refs 7, 31), suggesting that stGRNs can indirectly relay sweet taste information to the GNG. Remarkably, sugar-induced suppression of walking is controlled by the stGRNs (Fig. 7 and Supplementary Fig. 8). The projection of the stGRNs terminates exclusively in the VNC (Fig. 3), and may have better access to the VNC circuits that directly control locomotion. As we did not find direct connections between stGRN terminals and leg motor neurons, the stGRNs may suppress locomotion using unidentified local circuits. Because detection of food promotes multiple behaviours, functional specialization in tarsal GRNs is an effective way to coordinate the initial responses. We propose that stGRNs are tuned to suppressing competing behaviours such as locomotion, while atGRNs promote the change to the feeding state through PER (Fig. 8).

The importance of tarsal GRNs in sugar choice (Fig. 2) fits well with the fact that legs are typically the first appendage to contact food. Recent physiology studies identified hypersensitive sweet taste GRNs in the fifth tarsal segment^4^,^25^. According to their innervation of taste hairs, these are likely the atGRNs (Fig. 4c,d). Arrangement of GRNs that are sensitive and important for appetitive behaviour in the ventral tip of the tarsus is an appropriate cellular configuration, given the maximal accessibility to food. The direct projection of the atGRNs to the GNG may further ensure rapid and efficient feeding initiation. Bees also have taste hairs with very high sugar sensitivity in their tarsi^32^ and antennae^33^, which are presumably the first organs to detect nectar. Another taste-driven behaviour, tapping of the female abdomen by male flies during courtship, involves GRNs specifically on the dorsal area of the forelegs^34^. Taken together, these examples suggest that optimization of taste hair position with respect to function is a general principle in insect gustation.

Tarsal GRNs are stimulated as soon as a fly steps on tastants and may therefore be important for early gustatory responses across taste qualities. In accordance, bitter GRNs in the legs, but not the proboscis, are required for aversion to a bitter chemical^15^. On the other hand, GRNs in the labellum and pharynx come into play in later stages of feeding and may have distinct functions. In line with this idea, sweet taste receptor neurons in the LSO and ventral cibarial sense organ drive food choice in a longer-lasting (2 h) assay, presumably by prolonging ingestion^17^. Similarly, the GRNs in the labellum might be necessary to guide the mouth part to a food source more accurately or to open the labial palps with greater efficiency than that observed after tarsal stimulation. Because taste organs are differentially represented in the brain^35^ and send projections to discrete clusters in the CNS^7^,^14^, they most likely contribute to distinct aspects of feeding behaviour. Future studies focusing on fine neuronal manipulations, detailed characterization of appetitive behaviours and a mechanistic view of their interplay will promote understanding of the neuroethology of feeding.

## Methods

### Fly strains

The following transgenic strains of *D. melanogaster* in a *w*^*1118*^ background were used for crosses: *y w hsp70-flp; Sp/CyO; TM2/TM6b* (ref. 36); *UAS*>*CD2 y*^*+*^*>CD8-GFP* (ref. 24; original donor Gary Struhl); *w Gr5a-LexA; Bl/CyO; TM2/TM6b* (ref. 37); *w; LexAop-GAL80* in attP40 (gift from B. Pfeiffer and G. Rubin, Bloomington #32214); *w; Pin/CyO; UAS-mCD8::GFP* (ref. 38); *w;; UAS-Kir2.1::eGFP* (ref. 22), *y w LexAop-mCD8::GFP UAS-mCD8::RFP* (ref. 39; gift from B. Pfeiffer and G. Rubin, Bloomington #32229); *w; UAS-mCD8::RFP* (gift from Ilona Kadow); *w;; LexAop-rCD2::GFP* (ref. 26); *w; otd-nls-FLPo* (ref. 29); *w; Bl/CyO; tub>GAL80>* (Bloomington #38881); *w; Gr5a-GAL4/CyO; Dr/TM3* (ref. 8); *w; Sp/CyO; Gr43a-GAL4/TM3* (ref. 8); *w; Gr43a-GAL4* (ref. 26); *w; Gr61a-GAL4/CyO; Dr/TM3* (ref. 8); *w; Sp/CyO; Gr64a-GAL4/TM3* (ref. 8); *w; Sp/CyO; Gr64c-GAL4/TM3* (ref. 8); *w; Sp/CyO; Gr64d-GAL4/TM3* (ref. 8); *w; Gr64e-GAL4/CyO; Dr/TM3* (ref. 8); *w; Gr64f-GAL4/CyO; MKRS/TM2* (ref. 8); *w;; Gr64f-LexA* (ref. 26); and *white* (*w*^*1118*^). We used Canton S flies as the wild-type strain, but the appropriate generic controls (in *w*^*1118*^ background) for comparisons. All flies were kept at 25 °C and 60% relative humidity on standard cornmeal medium under a 14 h/10 h light/dark cycle.

### Immunohistochemistry

Brains, VNCs, proventriculi, uteri and proboscises of 2- to 8-day-old adult female *Drosophila* were dissected as previously described^40^, fixed for 45 min at room temperature in 4% formaldehyde in phosphate-buffered saline (PBS) with 0.1% Triton X-100 (PBS-Tx), washed with 0.1% PBS-Tx and stained using antiserum to green fluorescent protein (GFP; rabbit, 1:1,000, Invitrogen; or rat monoclonal 3H9, 1:100, Chromotek) and red fluorescent protein (RFP; rabbit 1:100, Clontech). Fixation and immunostaining was avoided for forelegs and some proboscis samples (Supplementary Figs 2 and 5); these were imaged immediately after dissection. To visualize synaptic neuropil regions, mouse monoclonal antibody for Synapsin^41^ (1:100, Developmental Studies Hybridoma Bank; Iowa City, IA) or rat monoclonal antibody for N-Cad (1:100, Developmental Studies Hybridoma Bank; Iowa City, IA) were used. For detection of primary antisera, Alexa 488-tagged goat anti-rabbit (1:1,000, Invitrogen), Alexa 488-tagged goat anti-rat (1:200, Invitrogen), Cy3-tagged goat anti-mouse (1:250, Jackson Immunoresearch), Cy3-tagged goat anti-rabbit (1:250, Jackson Immunoresearch), Cy3-tagged goat anti-rat (1:200, Jackson Immunoresearch) and Alexa 633-tagged goat anti-mouse antisera (1:250, Invitrogen) were used. Preparations were mounted in Vectashield (Vector; Burlingame, CA), 70% glycerol (Sigma-Aldrich) in PBS or 70% 2, 2-thiodiethanol (Sigma-Aldrich) in PBS, and imaged with either an Olympus FV-1000 (brains and VNCs) or a Zeiss LSM 780 confocal microscope including a T-PMT device (transmitted light detector for bright field images, for some tarsi and proboscises). To generate single-cell flp-outs, freshly eclosed flies carrying *hsp70-flp, UAS*>*CD2 y*^*+*^*>CD8-GFP* and *Gr64f-GAL4* were heat-shocked in a 37 °C water bath for 30 min and dissected 4–5 days later. All images were processed using Fiji software^42^.

### Behavioural experiments

Genetic crosses were raised at 25 °C. F1 progeny were transferred to fresh food vials and were allowed to feed for at least 24 h before starvation. Flies were subsequently starved in moistened vials until a mortality rate of roughly 20% was achieved. As different genotypes vary in starvation resistance, average starvation times ranged between 31 and 49 h. All flies were 3–7 days old at the time of the experiment. Testing times were distributed throughout the day to minimize effects of circadian rhythm on performance. Behavioural experiments were performed at 25.0±0.3 °C and 60–70% relative humidity.

*Sugar preference assay*. Mixed populations of males and females were tested for sucrose preference (Calbiochem) in a circular arena (⊘ 76 mm). Each half of the arena was covered with a semicircular piece of filter paper that had previously been soaked with either 350 μl of water or 350 μl of a 0-, 0.1-, 0.5-, 1- or 2-M sucrose solution; filter papers were subsequently allowed to dry. The walls of the arena were covered with Fluon (Fluon GP1, Whitford Plastics Ltd., UK) to prevent flies from climbing. After introduction, flies were allowed to choose between the two sides for 1 min. Fly behaviour was video-recorded from above (Canon EOS 500D). The videos were processed using Matlab and Fiji softwares. Automatic fly counting was done as previously described^43^, and the PI was calculated as:


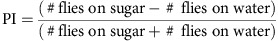


Pooled PI values (30–60 s) are presented for most experiments. To approximate the dependence of the sugar preference on starvation time and/or sucrose concentration, we fitted the following equation to the corresponding data:


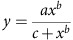


In this equation, *x* represents starvation time or sucrose concentration and *y* represents PI.

*Proboscis extension reflex*. Flies were briefly anesthetized under CO_2_. Female flies were selected and glued on their back on a coverslide with nail polish. Flies were allowed to recover in a humidified chamber for at least 1 h before the experiment. After recovery, flies were presented with water on their tarsi and were allowed to drink *ad libitum*. Unresponsive flies were discarded. After the flies stopped responding to water, sucrose solutions were presented on the tarsi in triplicate, from lowest to highest concentration. Water was given after each presentation to wash the tarsi and ensure that flies remained water-satiated throughout. PER was scored as 0 or 1 (0: no extension; 1: extension). To approximate the dose–response curve, we fitted the same equation as above. Labellar PER was performed as described elsewhere^27^.

*Olfactory learning*. Flies were trained and tested for immediate appetitive olfactory memory as previously described^28^,^44^. Odours (4-methylcyclohexanol and 3-octanol, diluted 1:80 and 1:100 in paraffin oil; Fluka, Germany) were presented in odour cups with a diameter of 14 mm. A learning index was calculated as the mean preference of two separate groups of reciprocally trained flies. In half of the experiments the first presented odour was rewarded and in the other half, the second presented odour was rewarded^45^. Aversive memory^45^ of starved flies using the same odours served as controls for intact locomotion and odour responses.

*Quantification of fly locomotion*. Videos were acquired as described above and analysed in Matlab. First, the region of interest (circular arena) was selected and coloured images were converted to greyscale. To distinguish flies from the background, images were binarized by applying a user-defined threshold. The binarized background (empty arena) was subtracted from all binarized images. Resulting images were eroded and dilated to remove noise^46^. A cluster of contacting pixels was labelled as one particle. For each particle, we computed its area, its centroid, the diagonal length of its bounding box and the eccentricity of an ellipse with the same second moments as the particle.

We calculated the likelihood for each particle representing a fly based on the diagonal lengths of the bounding box and eccentricity. Particles with low likelihood represented either flies for which the selected threshold was not appropriate, or multiple flies that were merged because of close proximity. We estimated the number of flies in low-likelihood particles by dividing their area with the average area of the high-likelihood particles. Then, we re-adjusted the threshold using an iterative process, until the number of particles in the region matched the estimated number of flies. If this did not occur after forty iterations, we excluded those low-likelihood particles. Ellipses were fitted to all particles as described elsewhere^47^.

We then used a closest-neighbour approach to identify individual flies in every pair of consecutive frames. First, we defined a pair set as all the possible pairs of ellipses between the two frames and computed the distances between the centroids of all ellipse pairs. Second, we determined the smallest distance and identified the corresponding ellipses as a pair. Third, we eliminated that ellipse pair from the pair set. We repeated the second and third steps until the pair set was emptied. We set the maximum changes in position and angle of paired ellipses in consecutive frames to ∼7 mm and 90°, respectively. Using this information, the change in position, angle, linear velocity, angular velocity and absolute angular velocity of each ellipse was computed. The Matlab script is available on request.

All computations were carried out on a parallel computer LX406Re-2, which consists of 68 nodes. Each node is equipped with a main storage of 128 GB and two groups of 12-core Intel Xeon processors E5-2695v2. The Matlab script outlined above was run on one node. In each node, parallel processing with automatic parallelization, Open Multi-Processing or Message Passing Interface can be operated up to 24 parallels. The maximum computing performance per node becomes 460.8 GFLOPS (Giga Floating-point Operations Per Second).

### Mathematical model of sugar preference

We assigned flies in the sugar preference assay (Fig. 1) to four states: free to walk on the sugar side (FS); free to walk on the water side (FW); feeding on the sugar side (S); and feeding on the water side (W) (Fig. 8a). Transitions between states were reversible and controlled by certain rates (constants *k*). The PI was obtained using:


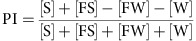


The number of flies in each state was obtained by solving the differential equations describing the transitions:


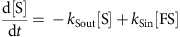



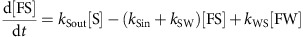



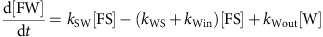



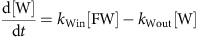


We assumed an equilibrium, solved the differential equations and substituted into the PI formula, thereby obtaining:


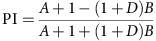


Here ‘sugar affinity' *A*=*k*_Sin_/*k*_Sout_, ‘speed ratio' *B*=*k*_SW_/*k*_WS_ and ‘water affinity' *D*=*k*_Win_/*k*_Wout_. Because we never observed flies feeding on the water side, we set the water affinity at an arbitrarily low level (*D*=0.001). We consequently examined the relative contributions of sugar affinity and sugar-induced locomotion suppression on PIs by varying parameters *A* and *B* (Fig. 8b).

To predict PIs for the *Gr5a-GAL4* and Gr(64f–5a) blockades (Fig. 8c,d), we first used data from genetic control experiments to determine values of *A* and *B* as functions of sucrose concentration. We reasoned that PER on stimulation of the tarsi with sucrose solutions recapitulated sugar affinity *A*. Therefore, *A* was derived as a function of sucrose concentration from the PER data as follows. First, a sigmoidal curve was fitted to the PER data using the equation:


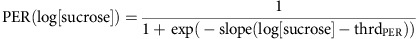


where ‘slope' and ‘thrd_PER_' are the parameters of the sigmoid function. Second, PER data were transformed to affinity using:


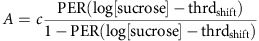


Here ‘thrd_shift_' and *c* are correction factors that account for the different conditions between the PER and sugar preference experiments (we acquired *c*=0.83 and thrd_shift_=3.1). The transformation produced a sugar affinity *A* between zero and infinity that is more appropriate for our model (*A*=0 when *k*_Sin_=0 and *A*=∞ when *k*_Sout_=0). The speed ratio B was calculated from the linear velocity data of genetic controls in the presence or absence of 2 M sucrose. Like sugar affinity, linear velocity was assumed to be a sigmoidal function of sucrose concentration. In addition, contributions of sugar affinity *A* and speed ratio *B* to sugar preference were quantified by simulating PIs of a theoretical sugar affinity mutant (*A*=*D*=0.001) and locomotion suppression mutant (*B*=1; Fig. 8e).

### Statistics

Data were evaluated using Prism software (GraphPad, San Diego, CA) as previously described^48^, employing Shapiro–Wilk and Bartlett's test. Data are presented as means±s.e.m. if they are normally distributed and have equal variances, and were tested with one-way analysis of variance and Bonferroni-corrected pairwise comparisons. Data are presented as medians, with the lower and upper error bars representing the first and third quartiles, respectively, if they are not normally distributed and/or variances are not equal. In that case, we applied the Kruskal–Wallis test and Dunn-corrected pairwise comparisons, the Mann–Whitney *U*-test or the Wilcoxon signed-rank test to check for statistically significant differences. An exception was made for PER data, which are presented as means±s.e.m. for ease of visualization, even though they are not normally distributed. However, we applied nonparametric statistics to check differences in the PER data as described above. Significance levels are indicated as follows: NS *P*>0.05; *P<0.05; **P<0.01; ***P<0.001.

## Additional information

**How to cite this article:** Thoma, V. *et al.* Functional dissociation in sweet taste receptor neurons between and within taste organs of *Drosophila*. *Nat. Commun.* 7:10678 doi: 10.1038/ncomms10678 (2016).

## Supplementary Material

Supplementary Information Supplementary Figures 1-8
