# Supplementary material for: Functional dissociation in sweet taste receptor neurons between and within taste organs of Drosophila
Source: Nat Commun. 2016 Feb 19;7:10678. doi: 10.1038/ncomms10678 (PMC4762887; doi:10.1038/ncomms10678)
Supplement: Supplementary Information — Supplementary Figures 1-8 [file ncomms10678-s1.pdf]

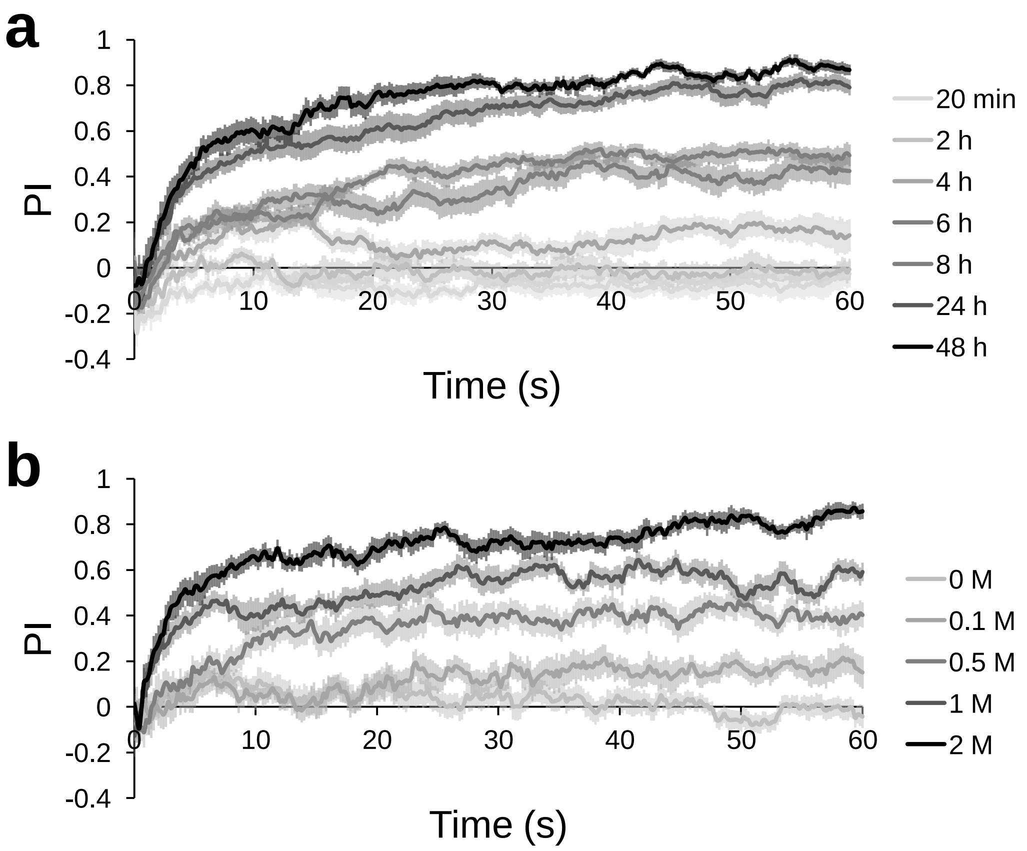

Supplementary Figure 1 | Time series of sucrose preference indices. (a) Effect of starvation on Preference Index (PI) time series. Increasingly longer starvation intervals (20 min–48 h; darker shades of grey) increase PI plateaus. *n* = 8–12 per starvation interval. (b) Effect of sucrose concentration on PI time series. Increasingly higher sucrose concentrations (0–2 M; darker shades of grey) increase PI plateaus. *n* = 11–15 per concentration. Results are means ± SEM.

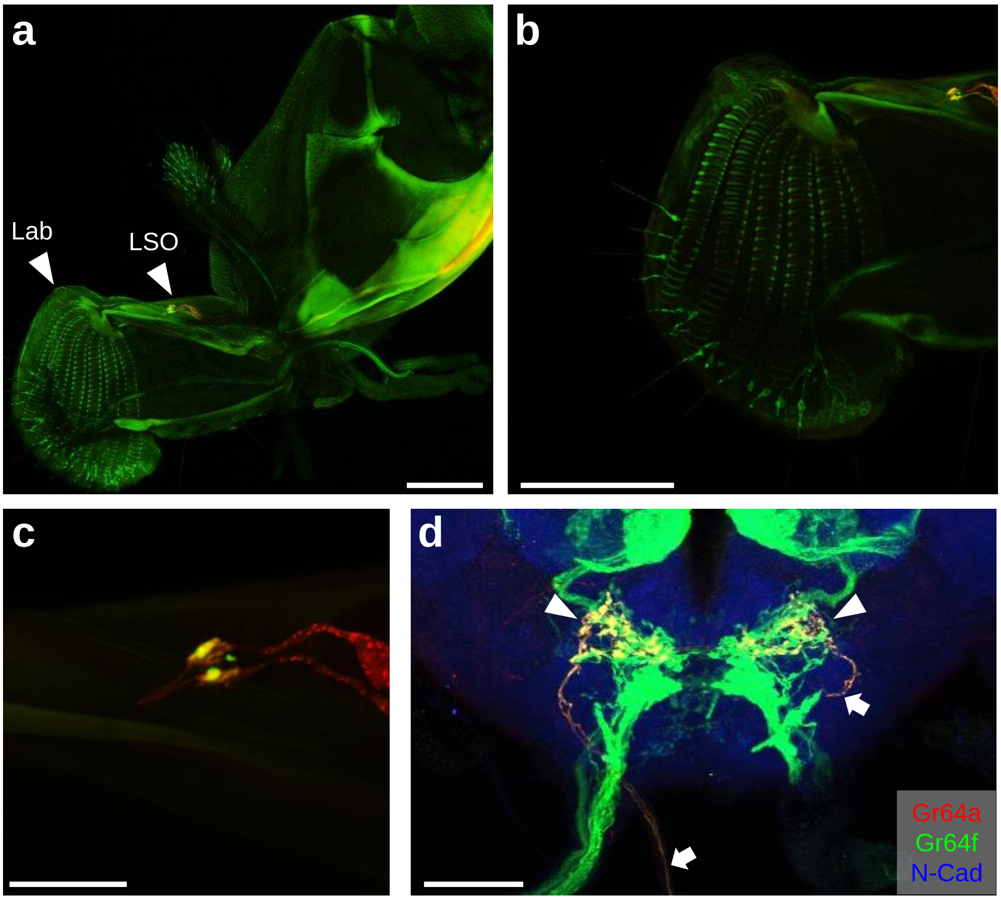

Supplementary Figure 2 | *Gr64a-GAL4* and *Gr64f-LexA* are co-expressed in the LSO. (a) Double-labelling of *Gr64a-GAL4* and *Gr64f-LexA* with *UAS-mCD8::RFP* (red) and *LexAop-rCD2::GFP* (green) in the proboscis. Labellum (Lab) and labral sense organ (LSO) indicated with arrowheads. (b) Blow-up of the labellum. Only *Gr64f-LexA* labels labellar GRNs (green). (c) Blow-up of the LSO. Both *Gr64a-GAL4* and *Gr64f-LexA* label a pair of LSO cells in each proboscis half (yellow). (d) Expression of *Gr64a-GAL4* (red) and *Gr64f-LexA* (green) in the gnathal ganglia. Neuropile was stained with an anti-N-Cad antibody (blue). Note that the pharyngeal nerve (arrows) and pharyngeal nerve terminals (arrowheads) are co-labelled by these lines (yellow). Partial projections, scale bars 100 μm (a, b), 20 μm (c) or 30 μm (d).

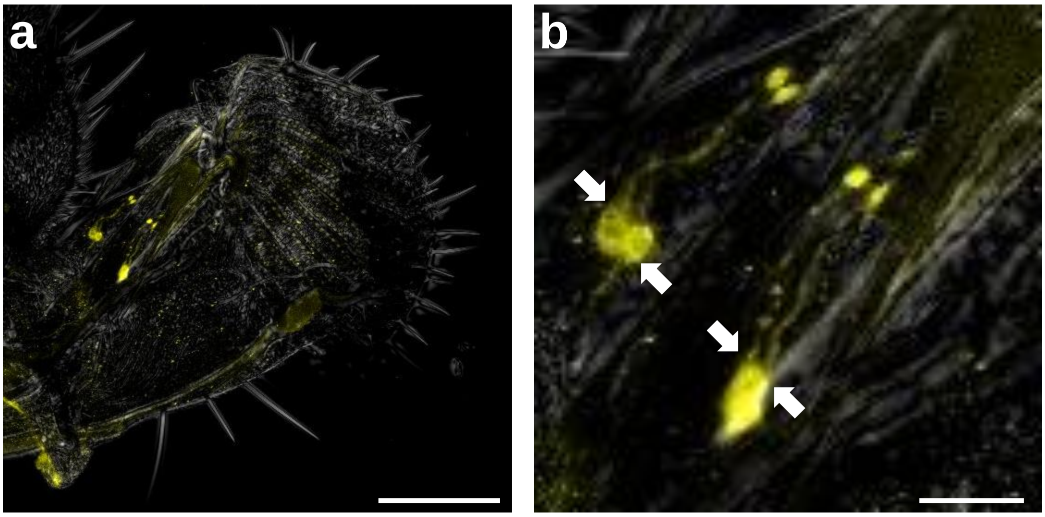

Supplementary Figure 3 | Expression pattern of Gr(64f–5a) flies in the labellum and the labral sense organ. (a) Expression pattern of Gr(64f–5a) flies in the proboscis. No expression in the labellum was detected, but labral sense organ cells were labelled. (b) Blow-up of the labral cense organ in (a). Note that four cells are labelled (arrows). Partial projections, scale bars 100 µm (a) or 20 µm (b).

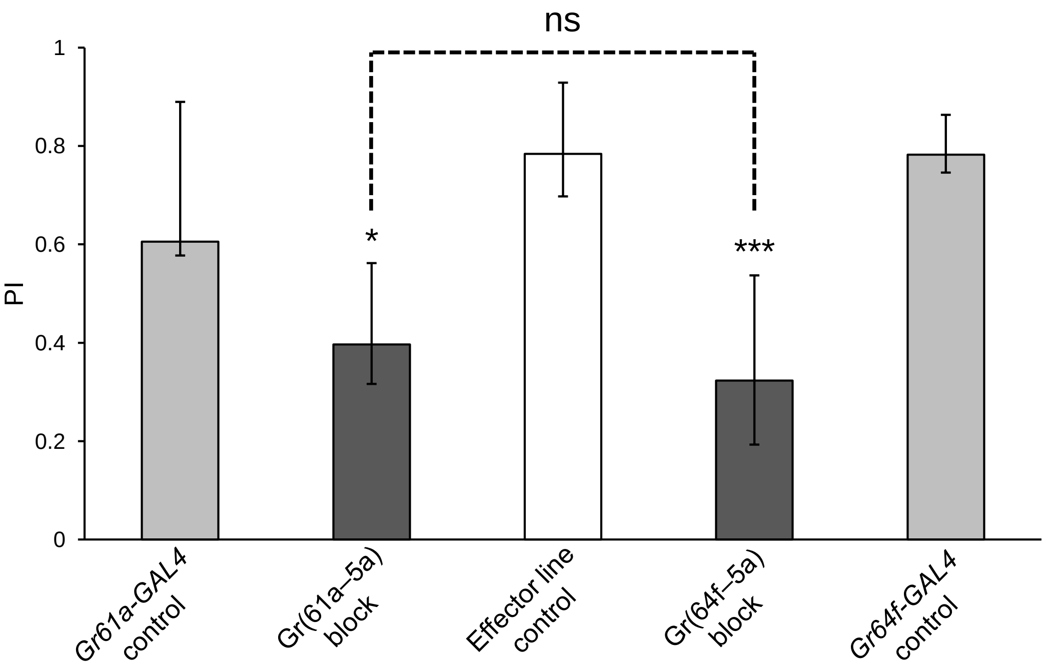

Supplementary Figure 4 | Gr(61a–5a) cells are required for sugar preference. Blocking Gr(61a–5a) cells significantly impaired 2 M sucrose preference index (PI) to the same extent as blocking Gr(64f–5a) cells. (Kruskal-Wallis test; Dunn's post test; * $P < 0.05$; *** $P < 0.001$; ns $P > 0.05$). $n = 15\text{-}21$ per group. Results are medians, with error bars indicating the first/third quartile. *Gr61a-GAL4* control *Gr61a-GAL4/+*, Gr(61a–5a) block *Gr5a-LexA/+; Gr61a-GAL4/LexAop-GAL80; UAS-Kir2.1/+*, effector line control *Gr5a-LexA/+; LexAop-GAL80/+; UAS-Kir2.1/+*, Gr(64f–5a) block *Gr5a-LexA/+; Gr64f-GAL4/LexAop-GAL80; UAS-Kir2.1/+*, *Gr64f-GAL4* control *Gr64f-GAL4/+*.

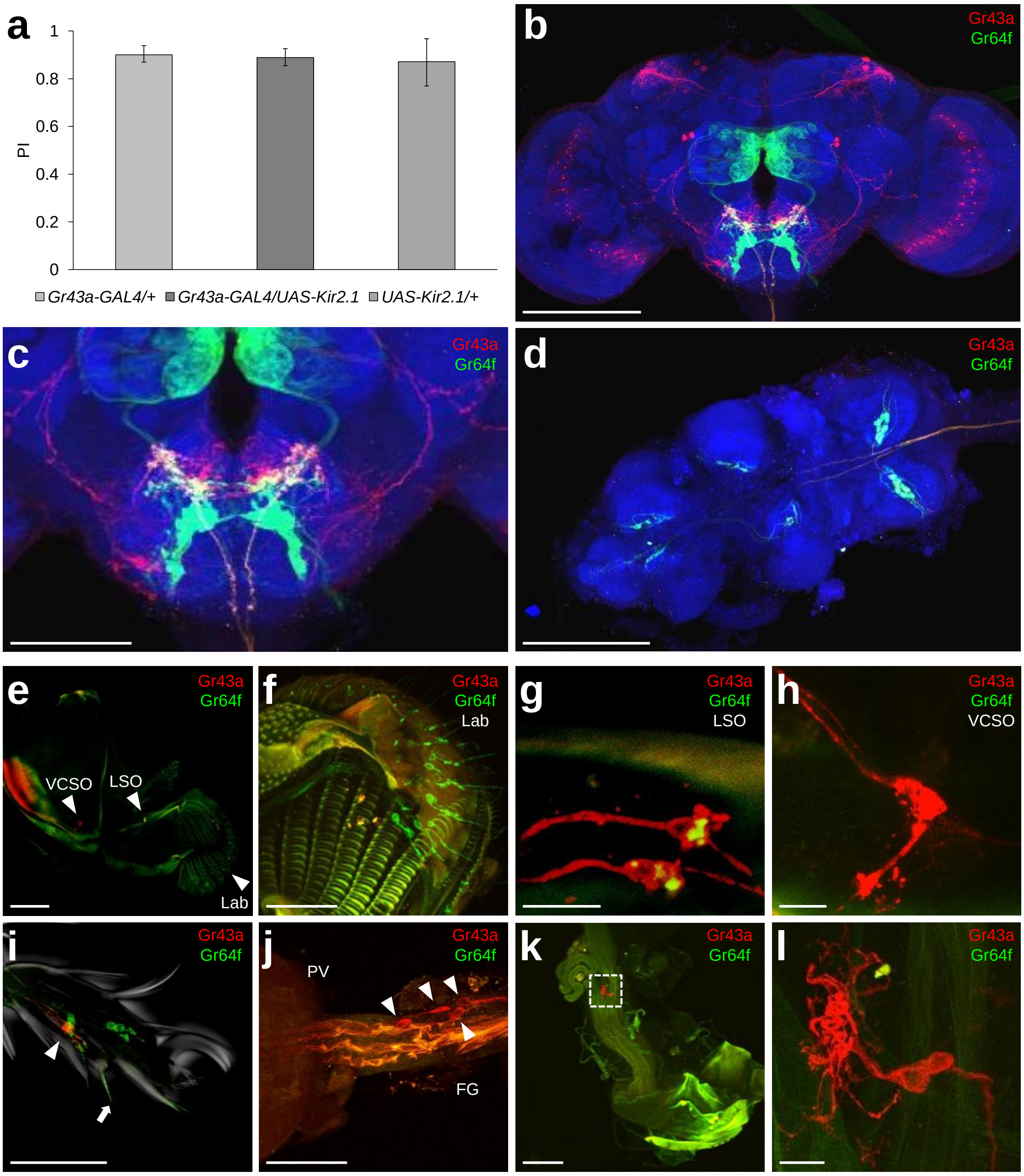

Supplementary Figure 5 | Behavioural and anatomical characterization of *Gr43a-GAL4*. (a) Electrically silencing GRNs in *Gr43a-GAL4* with constitutively active *UAS-Kir2.1* did not alter 2 M sucrose preference index (PI) compared to genetic controls (Kruskal-Wallis test; Dunn's post test; $P > 0.05$). $n = 11–12$ per group. Results are medians, with error bars indicating the first/third quartile. (b-l) Expression pattern of *Gr43a-GAL4* and *Gr64f-LexA* in the brain (b), gnathal ganglia (c), ventral nerve cord (d), proboscis (e), labellum (Lab, (f)), labral sense organ (LSO,

(g)), ventral cibarial sense organ (VCSO, (h)), fifth tarsal segment of the foreleg (i), foregut (FG)/proventriculus (PV) (j) and uterus (k-l). (*UAS-mCD8::RFP*, red; *LexAop-rCD2::GFP*, green; N-Cad, blue). Note that *Gr43a-GAL4* labels cells in addition to those covered by *Gr64f-LexA* in the brain, VCSO, FG/PV and uterus. Partial projections, scale bars 100 µm (b, d, e, k), 50 µm (c, f, i, j) or 10 µm (g, h, l).

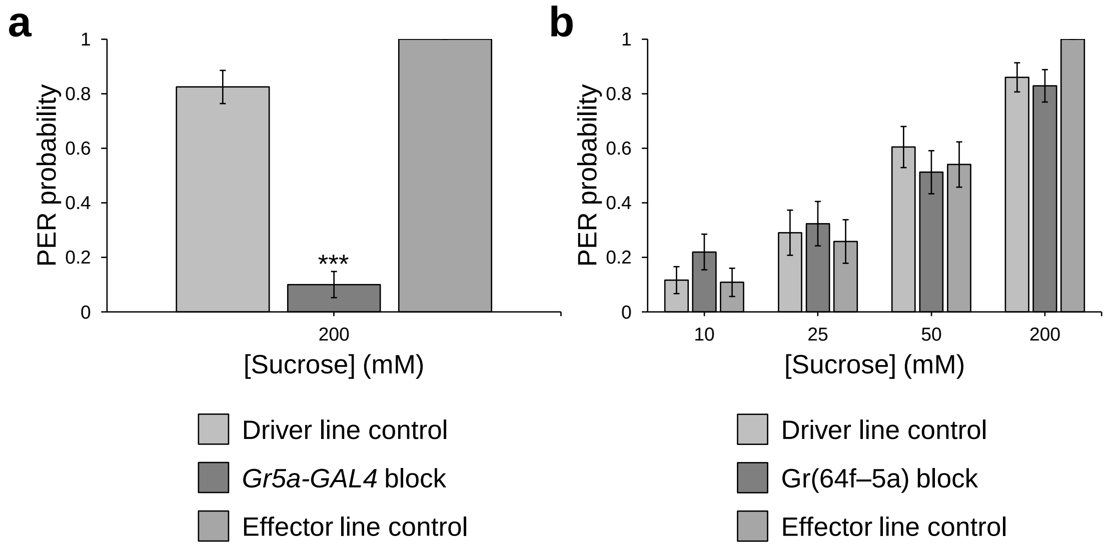

Supplementary Figure 6 | Labellar proboscis extension reflex is differentially affected by blocking subsets of sweet taste receptor neurons. (a) Labellar proboscis extension reflex (PER) with 200 mM sucrose was abolished when the gustatory receptor neurons in *Gr5a-GAL4* were blocked (Kruskal-Wallis test; Dunn's post test; *** $P < 0.001$). PER was indistinguishable from zero (Wilcoxon signed-rank test, $P > 0.05$). $n = 40$ flies per group. (b) Labellar PER was unaffected when the gustatory receptor neurons in Gr(64f–5a) were blocked (Kruskal-Wallis test; Dunn's post test; $P > 0.05$). $n = 31–43$ flies per group. Results are means ± SEM.

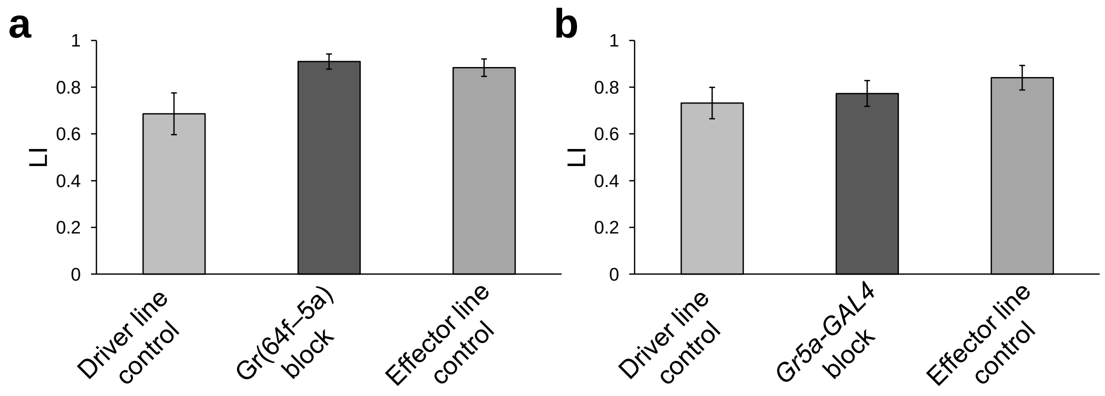

Supplementary Figure 7 | Short-term aversive memory remains intact upon blocking subsets of sweet taste receptor neurons. (a) Short-term aversive memory remained unaffected upon blocking Gr(64f–5a) cells (LI, learning index; one-way ANOVA; Bonferroni's multiple comparison test; $P > 0.05$). $n = 8$ per group. Driver line control *Gr64f-GAL4/+*, Gr(64f–5a) block *Gr5a-LexA/+; Gr64f-GAL4/LexAop-GAL80; UAS-Kir2.1/+*, effector line control *Gr5a-LexA/+; LexAop-GAL80/+; UAS-Kir2.1/+*. (b) Short-term aversive memory remained unaffected upon blocking *Gr5a-GAL4* cells (one-way ANOVA; Bonferroni's multiple comparison test; $P > 0.05$). $n =$ 8 per group. Driver line control *Gr5a-GAL4/+*, *Gr5a-GAL4* block *Gr5a-GAL4/UAS-Kir2.1*, effector line control *UAS-Kir2.1/+*. Results are means ± SEM.

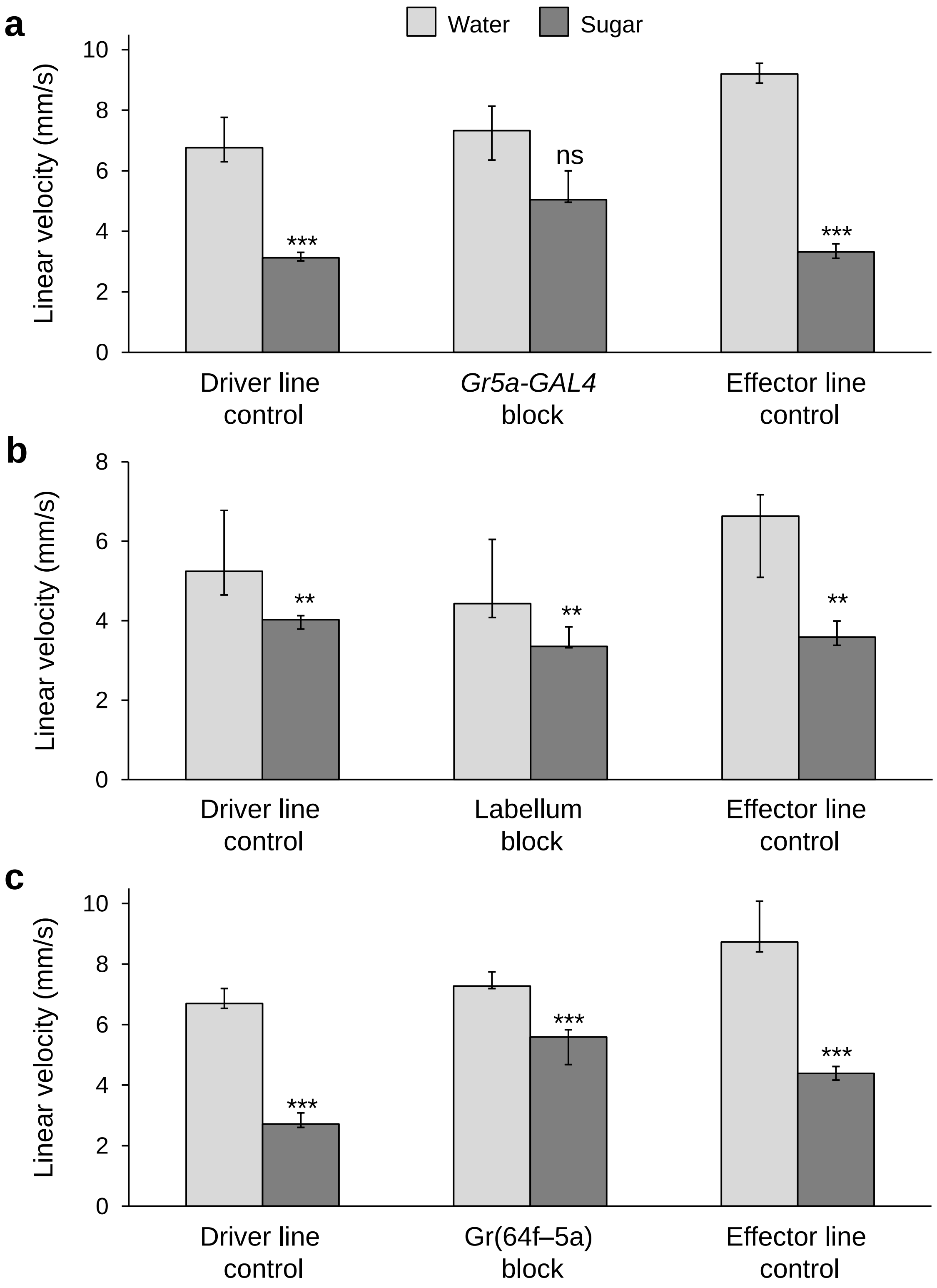

Supplementary Figure 8 | Segmental tarsal gustatory receptor neurons are required for sugar-dependent linear velocity suppression. (a) Average linear velocity of flies for the *Gr5a-GAL4* block and associated genetic controls between 30–60 s of the experiment in the absence (light grey, n = 15–16) or presence (dark

grey; n = 16–17) of 2 M sucrose. Sucrose significantly reduced linear velocity for genetic controls, but not for the experimental group (Mann-Whitney U tests; *** $P < 0.001$; ns $P > 0.05$). Driver line control *Gr5a-GAL4/+*, *Gr5a-GAL4* block *Gr5a-GAL4/UAS-Kir2.1*, effector line control *UAS-Kir2.1/+*. (b) Average linear velocity of flies for the labellum block and associated genetic controls between 30–60 s of the experiment in the absence (light grey, n = 13–14) or presence (dark grey, n = 13–14) of 2 M sucrose. Sucrose significantly reduced linear velocity for all groups (Mann-Whitney U tests; ** $P < 0.01$). Driver line control *Gr5a-GAL4/+; tub>GAL80>/+*, labellum block *Gr5a-GAL4/otd-nls-FLPo; tub>GAL80>/UAS-Kir2.1*, effector line control *otd-nls-FLPo/+; UAS-Kir2.1/+*. (c) Average linear velocity of flies for the Gr(64f–5a) block and associated genetic controls between 30–60 s of the experiment in the absence (light grey, n = 8–11) or presence (dark grey; n = 9–11) of 2 M sucrose. Sucrose significantly reduced linear velocity for all groups (Mann-Whitney U tests; *** $P < 0.001$). Driver line control *Gr64f-GAL4/+*, Gr(64f–5a) block *Gr5a-LexA/+; Gr64f-GAL4/LexAop-GAL80; UAS-Kir2.1/+*, effector line control *Gr5a-LexA/+; LexAop-GAL80/+; UAS-Kir2.1/+*. All results are medians, with error bars indicating the first/third quartile.
